# Milk Sickness

**Published:** 1855-03

**Authors:** 


### HALL'S JOURNAL OF HEALTH.

VOL. II.] MARCH, 1855. [NO. III.

### MILK SICKNESS,

Called, by some, *the Trembles*, is a disease prevalent in some parts of the West and South-West, and many have died with it. It is caused by drinking the milk of a diseased cow; a more fatal form of it arises from the use of butter or cheese, made from such milk, or from eating the flesh of any animal fattened with the milk, while the cow herself, nor the animal fattened with her milk, does not necessarily manifest symptoms of disease. One of the first questions of many seeking homes in the far west is, Has milk sickness ever been known here? Some of the finest lands in the world are without a market because the disease is in the neighborhood.

Soon after swallowing the milk, the person has thirst, nausea, swimming in the head, vomiting, fever, skin hot, eyeballs blood-shot, excessive debility, paralysis, oppression, stupor, hiccup, and death. In some cases, the heart beats with such violence as to strike the by-standers with horror, and even alarms the physician who has never witnessed it before.

The Legislature of one State at least, and perhaps of several others, has offered large rewards for the discovery of the thing which caused the cow to give such a deadly aliment. Kentucky offered a thousand dollars, but it has never been awarded, because various theories have been presented without a satisfactory quality of facts.

A recent visit to the West, and the usual reports of this one and that having died of milk-sickness, coupled with the fact of my connection with a health Journal, induce me to make a statement which I have never seen in print, and which I trust will do much good, if the newspaper press should give publication to the fact, and the farmers of the west would make a practical use of it. I will not take time here to meet objections to the statement I am going to make; the object is not argument, but a plain statement of what I consider a fact, which subsequent observation will establish in all time to come.

*Well fed cows never give milk-sickness.* I have revelled in the use of the most luscious milk, and the most delightful fresh butter for weeks together, in perfect fearlessness of milk-sickness, when several persons had just died of it on the next farm. The reason was, the cows were fed night and morning in winter with as much corn and meal as they wanted, and had sweet hay to eat during the day, and plenty of it; while, in the summer, they had fresh pasture and still something to eat of the slops of the kitchen at milking times, and knowing they would get something good, they never failed to come of their own accord, they thus literally "rolled in fat," summer and winter.

Some persons have attributed it to one vegetable, or weed, or grass; others to drinking from a certain spring, each locality having a different plant; these differences of opinion, together with the conceded fact, that it is not known on a well cultivated farm, are proofs, in my mind, of the truthfulness of the opinion which I have suggested.

Most persons who go far out west are poor, and soon become improvident. Very many study their ease, and how they can best remove the necessities of locomotion. To save chopping wood, for example, they take time by the forelock, and cut the bark off the tree for the space of a foot all around; the tree dies, and sooner or later, having become dry, the wind blows it down, and the limbs break into innumerable pieces, which are only to be picked up and put on the fire, cut and dried to hand.

The same improvident carelessness leads many to turn their cows like their pigs into the woods, to gather their own food, scarcely ever giving them a "nubbin" at milking; the result is, the cattle will eat closer than they otherwise would, especially in the fall of the year, when the grass is drying up, and the weeds have been wilted by frosts and being *eaten down*, or nibbed close to the ground, the roots many times give way, to which are attached sand and dirt; and I give it as my opinion, that this sand and dirt, taken into a system debilitated by scant feeding, causes the secretion of a milk which it is death to use. But whether it is the sand which attaches itself to the root of a close nibbed shrub or weed or grass, is not of the most practical importance; the two great facts already named, that a well fed cow has never been known by me to give diseased milk; and second, the general admission that milk-sickness is not known on a well cultivated plantation—for he who cultivates his land well, will always feed his cattle well—these two great facts are sufficiently instructive, and warrant the following advice:

Feed your cows well, and you will never be troubled with milk-sickness.

And when travelling in newly settled parts of the western country, or even through old settlements, never stop at a house where you see a poor cow at the door.

